# A Case of Male Goltz Syndrome

**DOI:** 10.1155/2012/728509

**Published:** 2012-10-18

**Authors:** Bhaswati Ghoshal, Subhrajit Lahiri, Debabrata Nandi

**Affiliations:** Pediatric Medicicne, Calcutta National Medical College and Hospital, Kolkata, India

## Abstract

We present the case of a boy with a clinical diagnosis of Goltz syndrome (focal dermal hypoplasia), a rare genodermatosis characterized by widespread dysplasia of mesodermal and ectodermal tissues. A 9-year-old male patient with Goltz syndrome presented with typical skin lesions along with progressive dimness of vision and mental retardation since birth. It is inherited in an X-linked dominant fashion and is normally lethal in male patients, and so very few male patients, like the index case, have been reported.

## 1. Introduction 

Focal dermal hypoplasia (FDH) (OMIM #305600) is a multisystem condition in which developmental defects of the skin are associated with ocular, dental, and skeletal abnormalities. Incidence is likely to be underestimated, as mildly affected subjects may go unrecognized. It is an X-linked dominant disorder; although this syndrome is normally lethal in male patients, approximately 10% of FDH cases are males, which is believed to be due to mosaicism for postzygotic mutations [1, 2]. We report a case of a male patient with Goltz syndrome. 

## 2. Case Report 

**Figure 1:**
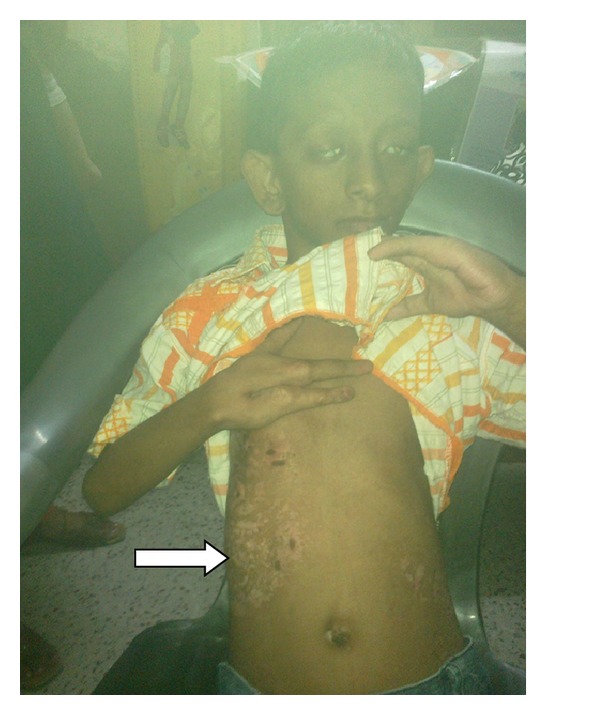
Hypopigmented streaks that follow Blaschko's lines.

**Figure 2:**
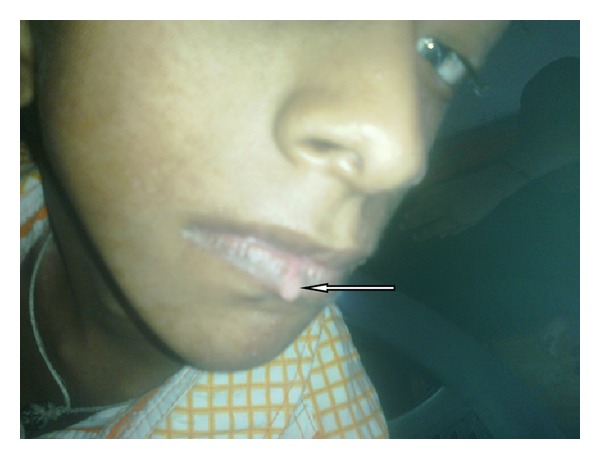
Raspberry-like papilloma present on lower lip.

**Figure 3:**
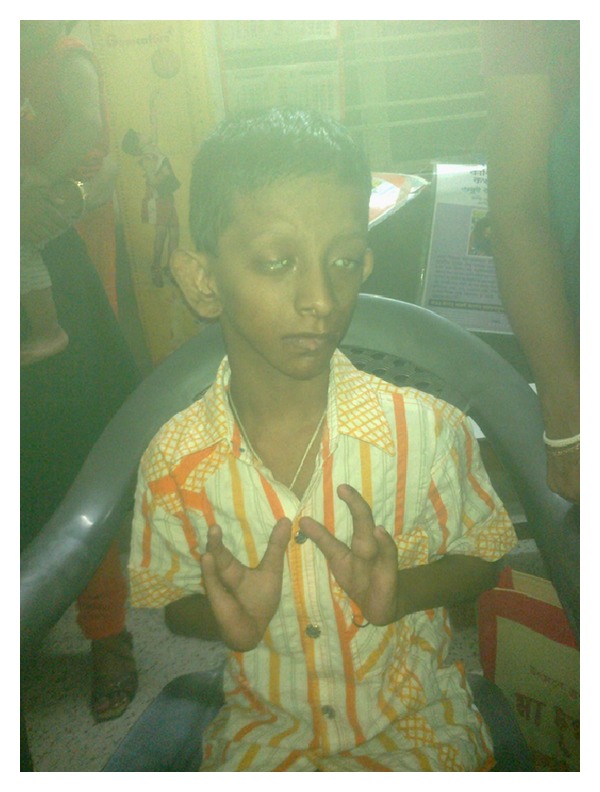
Lobster digits.

A 9-year-old male patient presented with asymmetric hypo-pigmented skin lesions along with progressive dimness of vision and mental retardation since birth. He was born normally, weighing 2.7 kg after 37 weeks of gestation with normal perinatal period, out of a nonconsanguineous marriage between phenotypically normal 25-year-old primipara mother and 30-year-old father. There is no family history of similar problems. Since birth, the child had a few asymmetrical linear streaks of atrophy and telangiectasia which have become hypopigmented streaks that follow Blaschko's lines (Figure 1). Raspberry-like papilloma is present in lower lip (Figure 2). There is generalized dryness of skin and occasional pruritus. Nails are dystrophic. Hair is sparse and brittle. He is slender with short stature and microcephaly. The chin is pointed, and the facial outline is triangular with protruding ears. Syndactyly and ectrodactyly of both hands are present (Figure 3). There is divergent squint with bilateral corneal opacity. There is also increased sandal gap. The presence of the lesions from birth and the association of linear streaks of atrophy and telangiectasia with soft fatty nodules and malformations of the digits confirm the diagnosis. Reconstructive surgery has been planned for rehabilitation of the patient. 

## 3. Discussion 

Goltz syndrome (FDH) involves tissues of ectodermal and mesenchymal origin. Findings vary from easily overlooked mild skin atrophy to severe limb deformity as in this case. Skin involvement has been present in all but 2 cases and is regarded as essential for the diagnosis [3]. 

Skin changes of FDH are the primary diagnostic features. There is linear, punctate, and streaky cribriform atrophy with telangiectasia as in this patient. The cribriform atrophy is marked by tiny ice pick-like depressions in the skin. These are distributed along the lines of Blaschko. Areas of thin to absent dermis are irregularly distributed, and the resultant herniations of fat appear as yellow-pink excrescence on the skin surface which are easily depressed. 

Papillomas that may be fleshy or vascular develop throughout life and favour the perigenital, perioral, intertriginous, and mucosal surfaces, in this case the lip. Papillomas in airway should be taken care of during intubation needed for general anaesthesia. 

Other dermatologic features include patchy alopecia, brittle or sparse hair, and palmar and plantar hyperkeratoses. Some individuals have had hyperhidrosis, and some have had aplasia cutis congenita, not present in this patient. Among the nondermatologic features are short stature, slender built, mental retardation, microcephaly, triangular face, protruding ear, and asymmetrical alae nasi. There may be also scoliosis, syndactyly, polydactyly, ectrodactyly, and facial clefting. Ocular defects include microphthalmos, anophthalmos, coloboma, strabismus, keratoconus, and corneal opacification. Intestinal malrotation and mediastinal dextroposition have been described in association with FDH [4]. 

Osteopathic stria may be found in radiogram of bones apart from giant cell tumors and osteochondromas. The diagnosis of FDH is relatively straightforward. Cribriform atrophy has been described in X-linked dominant Conradi-Hunermann syndrome (chondrodysplasia punctata), but ichthyosis is not a feature of FDH, and fat herniation is not part of Conradi-Hunermann. The streaky distribution of the atrophic lesions of incontinentia pigmenti (IP) is similar, as are the other system malformations, but the blistering, hyperkeratosis, and hyperpigmentation of IP are not found in FDH. In MIDAS syndrome (microphthalmia, dermal aplasia, and sclerocornea), the skin defects are limited to the head and neck; there is atrophy and scarring of the skin more similar to aplasia cutis congenita and not dermal atrophy alone. The disorders do share similar ocular abnormalities. 

The causative gene in this disorder has been identified as PORCN on chromosome Xp11.23 [5]. This is thought to encode an O-acetyltransferase involved in Wnt signaling which is important in ectodermal-mesodermal development during embryogenesis. To date, over 70 different mutations have been identified in patients with FDH, including six male patients [6]. A number of mutation-negative female patients have also been reported raising the possibility of the existence of genetic heterogeneity in this condition, or of mutations outside of the coding region [2]. Goltz syndrome in male patients is thought to result from somatic mosaicism; we believe this to be the case in our patient as well.
